# Identification of Placental Aspartic Proteinase in the Eurasian Beaver (*Castor fiber* L.)

**DOI:** 10.3390/ijms19041229

**Published:** 2018-04-18

**Authors:** Aleksandra Lipka, Grzegorz Panasiewicz, Marta Majewska, Lukasz Paukszto, Martyna Bieniek-Kobuszewska, Bozena Szafranska

**Affiliations:** 1 Department of Animal Anatomy and Physiology, Faculty of Biology and Biotechnology, University of Warmia and Mazury in Olsztyn, 10-719 Olsztyn, Poland; o.zamojska@gmail.com (A.L.); szafran@uwm.edu.pl (B.S.); 2 Department of Gynecology and Obstetrics, School of Medicine, University of Warmia and Mazury in Olsztyn, 10-719 Olsztyn, Poland; 3 Department of Human Physiology, School of Medicine, University of Warmia and Mazury in Olsztyn, 10-719 Olsztyn, Poland; marta.majewska@uwm.edu.pl; 4 Department of Plant Physiology, Genetics and Biotechnology, Faculty of Biology and Biotechnology, University of Warmia and Mazury in Olsztyn, 10-719 Olsztyn, Poland; pank24@gmail.com; 5 Department of Dermatology, Sexually Transmitted Diseases and Clinical Immunology, School of Medicine, University of Warmia and Mazury in Olsztyn, 10-719 Olsztyn, Poland; martyna.bieniek@uwm.edu.pl

**Keywords:** aspartic proteinases, beaver, PAGs, pepsinogen, placenta

## Abstract

Aspartic proteinases (AP) form a multigenic group widely distributed in various organisms and includes pepsins (pep), cathepsins D and E, pregnancy associated glycoproteins (PAGs) as well as plant, fungal, and retroviral proteinases. This study describes the transcript identification and expression localization of the AP within the discoid placenta of the *Castor fiber*. We identified 1257 bp of the *AP* cDNA sequence, encoding 391 amino acids (aa) of the polypeptide precursor composed of 16 aa signal peptide, 46 aa pro-piece, and 329 aa of the mature protein. Within the AP precursor, one site of potential *N*-glycosylation (NPS^119–121^) and two Asp residues (D) specific for the catalytic cleft of AP were identified (VLFDTGSSNLWV^91–102^ and GIVDTGTSLLTV^277–288^). The highest homology of the identified placental AP nucleotide and aa sequence was to mouse pepsinogen C (75.8% and 70.1%, respectively). Identified AP also shared high homology with other superfamily members: PAGs, cathepsins, and napsins. The AP identified in this study was named as pepsinogen/PAG-Like (pep/PAG-L). Diversified pep/PAG-L protein profiles with a dominant 58 kDa isoform were identified. Immune reactive signals of the pep/PAG-L were localized within the trophectodermal cells of the beaver placenta. This is the first report describing the placental AP (pep/PAG-L) in the *C. fiber*.

## 1. Introduction

Within the Rodentia, Castoridae is represented by the only two still-extant species, *Castor canadensis* (Cc), native in North America and *Castor fiber* (Cf) in Eurasia. Both species are characterized by their large body size and can be distinguished by karyological analyses indicating 40 or 48 chromosomes in Cc and Cf, respectively [1,2]. Taxonomically, previous multi-gene studies have suggested that the Geomyoidea superfamily—including the pocket gopher species (Geomyidae) and the kangaroo rats and mice (Heteromyidae)—is the closest to both beavers [3,4]. However, the analyses of mitochondrial DNA revealed squirrels (*Anomalurus*) as being the most closely related to beavers [5]. A recent study [6] confirmed the relationship between beavers (Castoridae) and kangaroo rat-related species (Geomyoidea) within a significantly supported mouse-related clade (including Myodonta, Anomaluromorpha, and Castorimorpha).

The Cf was widespread in Europe and Asia at the beginning of the 20th century, although over-hunting drastically reduced the population and range of this species. Ongoing conservation has prevented the beaver population from declining again and, as a result, the Cf has been classified by the International Union for Conservation of Nature as the Least Concern category [7].

Each mammalian placenta type is a functional connection between a mother and fetus. Beaver placenta has been classified as a discoid form with a hemochorial interface [8]. Since the placenta is a key component in understanding pregnancy regulation, characterizing crucial genes for placental development can serve as a novel basis for identifying mechanisms underlying effective reproduction in the Cf.

The multiple placental aspartic proteinases (AP) consist of orthologous genes evolved from duplication and adaptive diversification of an ancestor (pro-gene) in various eutherians [9]. Depending on the species classification, chorionic products reported in placentas of artiodactyls are named as the pregnancy-associated glycoprotein family (PAGs), whereas in non-artiodactyla species they are named PAG-Like (PAG-L) [9]. PAG-L are encoded by only one or two genes, while PAGs are encoded by a large multi-gene family [9,10]. Both PAG and PAG-L belong to the placental aspartic proteinase superfamily, which also includes various enzymes such as pepsins and several other lysosomal enzymes [9,10]. Among the AP superfamily, *PAG/PAG-L* is a large group of structurally-conserved genes encoding secretory chorionic products classified into two subfamilies, catalytically active and potentially inactive, although not yet examined in the Cf.

All members of the AP superfamily possess a bilobed structure with a cleft capable of binding short peptides. In vitro studies also revealed multiple secretory PAG isoforms produced by chorionic explants of domestic and wild species [11,12]. Radio-receptor studies revealed the potential physiological importance of PAG family products as various chorionic signaling ligands interacting with gonadal and extra-gonadal gonadotropin receptors of cycling pigs and cows [13] or early pregnant pigs [14].

Many purified native or several recombinant proteins required for anti-PAG sera production have led to the establishment of various diagnostic pregnancy tests based on PAG detection in maternal blood or milk by RIA (Radio Immuno Assay) and ELISA (Enzyme-Linked Immunosorbent Assay) analyses [9]. These PAG tests can be used to detect abnormalities during pregnancy in cattle [15,16] and to predict miscarriage after embryo transfer [17]. Since the varying PAG concentration depends on the number of healthy embryos/fetuses, it is higher in females with multiple, rather than single pregnancies, and can also differ due to the fetal sex and breed in many domestic and some wild ruminants [9].

It is hypothesized that the PAG-L family may also be expressed within the discoid placenta of the Eurasian beaver. Therefore, the objective of this study was to identify the existence of PAG-L/AP in the Cf: (1) in the placental transcriptome; (2) in the placental proteome including (a) protein profiles, and (b) cellular localization during advanced pregnancy.

## 2. Results

### 2.1. Identification of the AP Sequence in Placental Transcriptome of the Beaver

The RNA-seq generated a total of 160,784,163 raw reads and 149,440,078 clean reads (93%) were obtained after removing TruSeq adaptors and low-quality reads. TRINITY software enabled *de novo* assembly of 104,653 contigs. The reconstructed contigs were analyzed for their similarity to aspartic proteinases, which allowed the identification of a 1257 bp cDNA sequence of the placental *AP* transcript. Due to poor coverage of the *AP* cDNA during the RNA-seq, additional capillary sequencing was performed to evaluate and confirm the obtained data. Five pairs of homological primers used for PCR amplification were used to obtain the entire *AP* cDNA sequence. Among the 35 electrophoresed, gel-out purified and sequenced *AP* cDNA amplicons, only 22 clear chromatograms (HQ range: 20–89.2%) were subjected to further steps.

**Figure 1:**
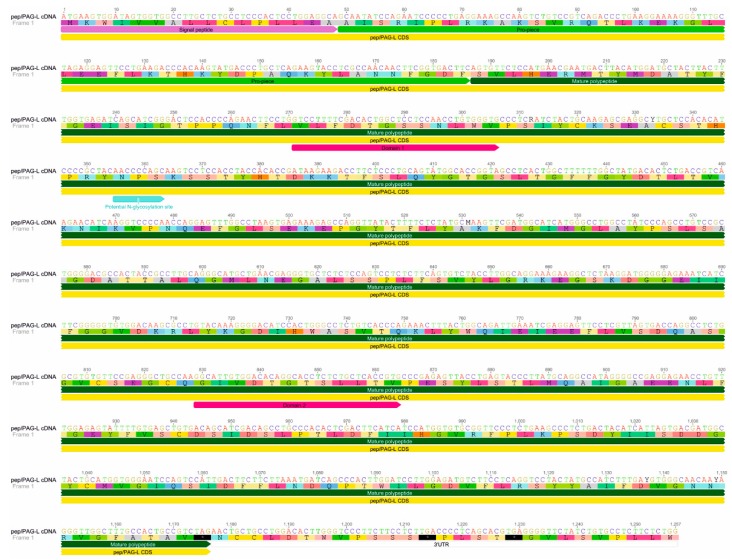
Beaver *pep/PAG-L* cDNA sequence. Identified 1257 nucleotides of *pep/PAG-L* cDNA (GenBank accession no. KU245742) coding 391 amino acids of polypeptide precursor. Signal peptide, pro-piece, mature polypeptide, domain 1 and 2 creating an active site, and a potential *N*-glycosylation site, were indicated.

Among the identified placental 1257 bp of *AP* cDNA sequence (GenBank accession no. KU245742), 1173 bp were determined as CDS (coding DNA sequence) and 86 bp as 3′ UTR (untranslated region) (Figure 1). A megablast of the *AP* sequence showed 77–95% homology with pepsinogens (pep; query cover 40–100%) identified in various species (e.g., North American beaver (*Castor canadensis*), kangaroo rat (*Dipodomys ordii*), golden hamster (*Mesocricetus auratus*), Philippine tarsier (*Tarsius syrichta*), Chinese hamster (*Cricetulus griseus*), killer whale (*Orcinus orca*), minke whale (*Balaenoptera acutorostrata*), bonobo chimpanzee (*Pan paniscus*), and sperm whale (*Physeter catodon*)).

Pair-wise alignment (GENEIOUS R7) of the *AP* cDNA sequence with selected AP members, known mainly in the mouse, showed the highest identity: 75.8% with pepsinogen C (GenBank accession no. NM_025973.3), 58.8% with porcine *PAG2* (pPAG2; GenBank accession no. L34361.1); 57.8% with pepsinogen F (GenBank accession no. AF240776.1); 56.8% with cathepsin D (GenBank accession no. NM_009983), and 55.5% with renin (GenBank accession no. J00621.1). Due to the nucleotide sequence homology and the placental expression, the *pep/PAG-L* name was given to the AP identified in the Eurasian beaver placenta.

**Figure 2:**
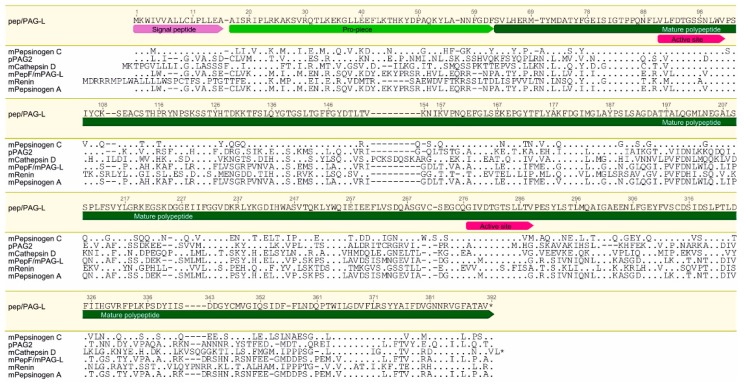
Comparison of the beaver pep/PAG-L polypeptide precursor amino acid sequence to representatives of the aspartic proteinases superfamily. Signal peptide, pro-piece, mature polypeptide, domain 1 and 2 creating an active site were indicated. Identical aa are dotted, omitted aa are dashed and stars indicate stop-codon. Abbreviations: m—mouse; p—porcine.

The identified *pep/PAG-L* cDNA encodes 391 amino acids (aa) of a polypeptide precursor sequence that revealed (BLSM62): 70.1% aa identity and 84.2% positive aa with pepsinogen C; 43.6% aa identity and 59.7% positive aa with pPAG2; 42.4% identity of aa and 57.7% positive aa with cathepsin D; 40.4% aa identity and 57.4% positive aa with pepsinogen F; and 37.4% aa identity and 54.4% positive aa with renin (Figure 2).

The theoretical molecular mass of the in silico identified pep/PAG-L protein was estimated for 43.392 kDa and isoelectric point for 7.99. Signal peptide (SP) was predicted for 16 aa in length (Figure 2). The identified SP aa sequence of the pep/PAG-L shared the highest similarity with SP of the mouse pepsinogen C, although it varied with the PAG family in different species (Figure 2). Additionally, alignment of the various APs enabled prediction of the 46 aa in length of a pep/PAG-L blocking peptide (17–62 aa) that shared higher homology with human pepsinogen C than with the PAGs (Figure 2). Therefore, a putative cleavage position was predicted between GDF^60–62^ of the blocking pro-piece and SVL^63–65^ of the mature pep/PAG-L (Figure 2). Within the entire 391 aa of the pep/PAG-L precursor sequence, two Asp residues (D), specific for the catalytic cleft of AP were predicted at positions 94 aa in the N-terminus (VLFDTGSSNLWV^91–102^) and 280 aa in the C-terminus (GIVDTGTSLLTV^277–288^; Figure 2). The sequence of the N-terminal domain of pep/PAG-L is identical to human and mouse pepsinogen C and very homologous to many other Aps; however, in the C-terminal domain, aa substitutions are more frequent, but the pep/PAG-L still shares relatively high homology with various AP members (Table 1). In addition, only one site of potential *N*-glycosylation (NPS^119–121^; Figure 1) was predicted in the pep/PAG-L precursor.

### 2.2. Identification of Placental pep/PAG-L Isoforms

**Figure 3:**
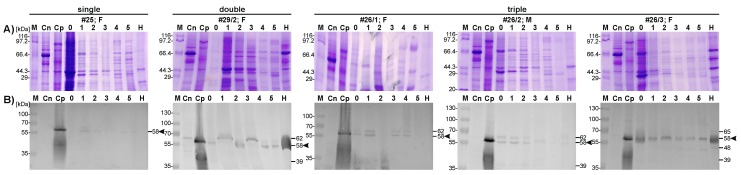
Detection of the beaver pep/PAG-L produced in vitro by beaver placental explants. (**A**) secretory and cellular proteins (10 µg/sample) separated by SDS-PAGE and stained by Coomassie Brillant Blue. (**B**) Western analysis with anti-porcine PAG-P polyclonals (1:300). Abbreviations: #—animal code; Cn—negative control (endometrial proteins of cycling pigs); Cp—positive control (porcine placental proteins); F—female fetus; M—male fetus; fractions (1–5) of secretory proteins harvested up to 209 h during in vitro cultures; 0—pre-culture; media collected after: 1—6 h, 2—18 h, 3—30 h, 4—42 h, 5—54 h of culture; H—cellular proteins; M—molecular marker. Note: arrowshead indicate dominant isoforms of the pep/PAG-L.

Western analysis permitted the identification of a dominant 58 kDa isoform of the pep/PAG-L proteins expressed in beaver placenta, despite the fetal sex and the multiplicity of gestation (Figure 3). Diversified profiles of the pep/PAG-L were identified for secretory proteins produced in vitro during the first 54 h (0–5 fractions) and for cellular proteins (H) isolated from various placentas (single, double, and triple).

In a female bearing one female fetus (#25 F), the dominant pep/PAG-L isoform was 58 kDa, while in a female bearing three fetuses, three various isoform profiles were identified: 58 and 62 kDa for a female fetus (#26/1 F) and 39, 58, and 62 kDa for a male fetus (#26/2 M). The fourth profile was identified in the placenta of a female fetus with 39, 48, 58, and 65 kDa isoforms (#26/3 F). The fifth profile of 39, 56, and 62 kDa isoforms was identified in the placenta of a male fetus from another female bearing two fetuses (#29/2 M).

### 2.3. Placental Localization of the pep/PAG-L Cellular Expression

**Figure 4:**
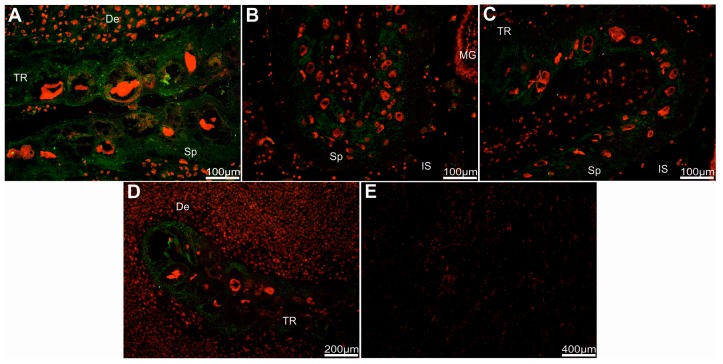
Detection of the beaver pep/PAG-L within subplacenta sections. Immuno-localization of pep/PAG-L signals within subplacenta sections, identified by htdF-IHC with anti-pPAG-P (**A**) or anti-Rec pPAG2 polyclonals (**B**–**D**), visualized by goat anti-rabbit IgG-conjugated with Alexa 488 dye (A488; green) among all placental cells with nuclei stained by propidium iodine (red). (**E**) Negative control with omitted primary antibodies and nuclei stained by propidium iodine. Abbreviations: De—decidua; TR—trophectoderm; Sp—spongiotrophoblast; IS—intervillous space; MG—maternal gland.

**Figure 5:**
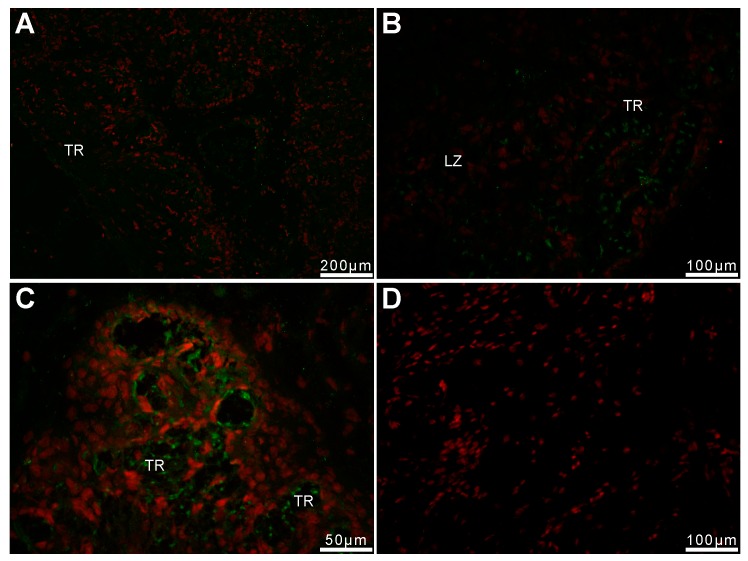
Detection of the beaver pep/PAG-L within labyrinth sections. Immuno-localization of pep/PAG-L signals within the labyrinth sections, identified by htdF-IHC with anti-pPAG-P (**A**,**B**) or anti-Rec pPAG2 polyclonals (**C**), visualized by goat anti-rabbit IgG-conjugated with Alexa 488 dye (A488; green) among all placental cells with nuclei stained by propidium iodine (red). (**D**) Negative control with omitted primary antibodies and nuclei stained by propidium iodine. Abbreviations: TR—trophectoderm; LZ—labyrinth zone.

Strong immune-positive pep/PAG-L signals were found in trophectoderm cells (chorionic epithelium) of both analyzed regions, labyrinth, and subplacenta sections. Heterologous dF-IHC detection with anti-pPAG-P (Figure 4A and Figure 5A,B) or anti-Rec pPAG2 polyclonals (Figure 4B–D and Figure 5C) revealed the presence of pep/PAG-L within the placental cells of fetal origin.

Relatively stronger immune-positive signals of the pep/PAG-L were detected by the anti-pPAG-P rather than with the anti-Rec pPAG2 polyclonals. For anti-Rec pPAG2 polyclonals (for non-glycosylated pPAG2 recombinant antigen), the immune-positive pep/PAG-L signals were not observed in the maternal part of the placenta. Distinct staining pattern of the anti-pPAG-P polyclonals revealed accumulated material within decidua secreted by adjacent trophectoderm. Noticeable pep/PAG-L signals were spread across all trophectodermal layers where spongiotrophoblast (Sp) were observed (Figure 4). There was increased intensity of the pep/PAG-L signals located near the trophoblastic cells, suggesting their secretory activity. In the labyrinth part of the placenta, the pep/PAG-L signals were detected in trophoblastic tubules (Figure 5). Groups of trophectodermal cells were located in close contact and formed lobules as larger fetal cores that resembled syncytial-like structures (Figure 5A).

## 3. Discussion

This is the first study identifying the AP superfamily member in the Eurasian beaver placenta, named pep/PAG-L. Diversified profiles of the cellular and secretory pep/PAG-L forms (kDa) were immuno-detected. Placental expression was localized within trophectodermal cells (chorionic epithelium). To date, this is the first study describing the component of molecular mechanisms involved in proper implantation, placenta development, and further embryo-maternal communication in the beaver.

The presently-identified and deposited 1257 bp *pep/PAG-L* cDNA (GenBank accession no. KU245742) shares at least 55.5% homology with some members of the APs, especially with numerous vertebrate pepsins. The AP is a multi-genic family of duplicated paralogous gene groups widely distributed in various species [18,19,20]. In mammals, besides PAGs, cathepsin D, E, and renin, the major members of the AP superfamily are pepsinogens [21]. Pepsinogens, initially considered to be restricted to the stomach, have the potential for expression in various tissues of vertebrates, e.g., intestine, lung, and pancreas [19]. In lower vertebrates, progastricsin cDNA was also found in the ovary of trout [22]. Remarkably, in the stomachless pufferfish, pepsinogen mRNA is expressed in the skin and gill but not in the digestive organs, which suggests the acquisition of a novel function of pepsinogens during the evolution of vertebrates [23].

Phylogenetic analyses revealed the duplication of progene that resulted in multiplicity of the pepsinogens as well as in the emergence of the PAGs expressed in mammalian placenta [21]. To date, the nucleotide sequences of the *PAG* cDNAs have only been identified in a few wild eutherian species: zebra, white-tailed deer, water buffalo, American bison, wapiti, and giraffe (GenBank). Without a doubt, this is due to difficulties with the proper condition of placenta harvesting required for high-quality RNA and cDNA cloning. The numbers of *PAG-L* cDNAs vary between species and are multiple in cattle, sheep, goats, and pigs, while a single *PAG-L* cDNA has been cloned in the horse, zebra, mouse, and cat [9,10]. Among the numerous diversified *PAGs* in various species, some *PAG-L* cDNAs share relatively higher nucleotide homology to each other than to pepsinogens, whereas some other members, share 55% and 52–57% homology with pepsinogens and other *PAGs*, respectively [9,21,24]. The evolutionary origin of the PAGs [10,19,25,26] may suggest why the presently identified placental AP shares higher homology with pepsinogens rather than PAGs. Alternatively, it cannot be excluded that beaver pep/PAG-L and pepsinogen C are products of the same gene, which would suggest the acquisition of a novel function of gastricsin within the placenta. It should be mentioned that despite extra-gastric expression of pepsinogen C in such tissues as the prostate, pancreas, lung, liver, and brain, there are no expression data that would indicate placental expression of gastricsin (http://bioinfo.wilmer.jhu.edu/tiger/db_gene/PGC-index.html). Taking in consideration nucleotide sequence homology and the placental expression, the name pep/PAG-L was given to the AP identified in the Eurasian beaver placenta, but it can be expected that the given name will be specified once a whole reference genome of the *C. fiber* is established.

The identified 391 aa pep/PAG-L precursor (Figure 1 and Figure 2), composed of 16 aa signal peptide, 46 aa pro-piece, and 329 aa of mature polypeptide with N-terminus (SVL^63–65^), resembled other AP precursors. It should be mentioned that one potential *N*-glycosylation site (NPS^119–121^) was predicted within the pep/PAG-L aa sequence, but proline in the X position strongly disfavors N-linked glycosylation [27], thus it is unlikely that this position is glycosylated. The in silico analyses of the pep/PAG-L precursor (43.394 kDa; Ip = 7.99 pH) in the current study confirmed the PAG family diversity. Previously, entire aa sequences of various PAGs varying in length, molecular mass, and electrostatic properties (375–389 aa, 30–90 kDa, and 4.0–9.08 pI, respectively) were identified [9]. Among the known PAGs, 15 aa signal peptides as well as 33–38 aa pro-pieces are very conservative in various species [21,24,28,29]. However, pepsinogen precursors are composed of 15–16 aa signal peptides, 42–46 aa activation segments, and 321–332 aa of mature proteins [19,30]. Moreover, high *N*-glycodiversity (up to seven *N*-glycosylation sites) is very common in the PAGs [9]. Post-translational glycosylation also occasionally occurs in pepsinogens, but there are generally no more than two *N*-glycosylation sites [30,31], similar to the pep/PAG-L (Figure 1). Thus, it seems that the pep/PAG-L precursor is more homologous with pepsinogens than PAGs. This is a novelty within the PAG-L family, so far, all identified PAGs have also shared relatively high homology with pepsinogens as well as other AP representatives, but generally were most homologous with other PAGs.

The pep/PAG-L precursor was classified as catalytically active due to the conserved sequences (two aspartic acids) located in two domains (N- and C-terminal lobes), creating the substrate binding cleft existing in all APs (Figure 1, Table 1). Similarly, the pepF/PAG-L identified in the mouse [26], horse, zebra, and cat are also active APs [24]. However, in species with multiple PAG family members, e.g., in the pig and the white-tail deer, active as well as potentially inactive forms are known [28,32,33]. Loss of catalytic activity is caused by various mutations generating multiple aa substitutions within two domains creating the binding sites of many PAGs [9,21,34,35]. Presumably, differences between the features of the PAG-L family in species of the Rodentia (beaver or mouse) or the Artiodactyla order (e.g., pig, cattle or deer) result from the different placental types of those taxa.

Our Western-blotting (Figure 3) with anti-pPAG-P polyclonals identified diversified placental protein profiles of heterogeneous native pep/PAG-L forms (38–62 kDa). Similar to cattle and pig, placental explants release various PAG forms, 45–85 and 43–70 kDa, respectively [11,36]. Such heterogeneity of bovine PAGs is caused by tetra-antennary glycans [37]. Three PAG isoforms (72, 74, and 76 kDa) also occur in the American bison placenta [38]. Among the Cervidae, various PAG isoforms exist, 33–55 kDa in the white-tailed deer [33], 39–62 kDa with diversified NH2-terminals in the fallow deer [39], although the dominant 55 kDa fraction is characteristic for different pregnancy stages (50–200 dpc) in the European moose [40]. Thus, the variety of the PAGs may reflect different functions as pregnancy advances, as a local suppressive factor influencing the feto-maternal barrier by modulation of the maternal immune system [10] and as chorionic ligands for the luteal and uterine gonadotrophin receptors of cycling and pregnant animals [13,14,41]. Previously, diversified profiles of the PAGs (e.g., in pigs and European bison) were an effect of multiple glycosylation [12,42]. In this study, diversified pep/PAG-L profiles were also identified, although only one site of potential glycosylation was predicted within the amino acid sequence of the pep/PAG-L precursor. This may suggest that diversified pep/PAG-L profiles are caused not by glycosylation, but by other post-translational modifications. Additionally, it is also possible that the immunodetected pep/PAG-L isoforms originated from alternative splicing events.

Localization of the pep/PAG-L cellular expression, detected by the heterologous dF-IHC (Alexa 488/PI), identified signal distribution within chorionic cells of the beaver discoid placenta (Figure 4 and Figure 5). Localization of the pep/PAG-L expression is difficult to compare because similar analyses have not been performed in other species with discoid placenta. To date, cellular PAG expressions have only been localized in some species of the Artiodactyla order with cotyledonary: cattle [43], white-tailed deer [33], bison [44], moose [40]; or diffuse placenta: pig [45] and alpaca [46]. Localization of the PAGs expression is also known in one- and two-humped camels that develop placenta more similar to diffuse placenta during early pregnancy, but during later stages of pregnancy, the placenta resembles a cotyledonary-type [46]. In the European bison, EbPAGs are expressed in developed placentomal regions, in the apical (uterine-directed) area of cotyledonary villous folds, and higher expression occurs in the cytoplasm of enlarged trophectodermal cells [44]. In white-tail deer placentomes, wtdPAGs are predominantly expressed in bi-nucleated cells [33]. In the moose, the signal intensity of the AaPAG-Ls is related to placentome growth during pregnancy and is stronger in secretory granules of trophectodermal cells located in the vicinity of the uterine compartments [40]. Thus, the PAG-L family retains characteristic chorionic expression features despite the morphological and developmental differences of various placenta types. The localization of the pep/PAG-L expression, as other PAGs, is restricted only to embryo-originated trophoblastic and trophectodermal cells due to pregnancy stages. However, this is the first report concerning pep/PAG-L family signals localized near trophoblastic cells. This may suggest that pep/PAG-L can be secreted directly into the maternal blood space. To date, the PAGs have been localized only within epitheliochorial placentas [12,28,40,44,46,47]. In ruminants, multiple granules of binucleated cells are released close to the maternal circulation, while the trophectodermal barrier to other feto-maternal exchange is maintained [43,48].

## 4. Materials and Methods

### 4.1. Ethics Statement

Beavers were captured and sacrificed with the permission of the Regional Directorate for Environmental Protection in Olsztyn (RDOS-28-OOP-6631-0007-638/09/10/pj), confirmed by the III Local Ethical Commission for Experiments on Animals at Warsaw University of Life Sciences (11/2010). The protocol of this study was approved (14 December 2011) by the Local Ethical Commission for Experiments on Animals at the University of Warmia and Mazury in Olsztyn (UWM/111/2011/DTN for the project 2012/07/N/NZ9/02050).

### 4.2. Tissue Harvesting

Beavers (from north-eastern Poland) were captured in cages by members of the Polish Hunting Association and transported to the Research Station of the Polish Academy of Sciences in Popielno. All used animals had a good nutritional status (body condition) and were healthy. The beavers were anesthetized with an injection of 2.5 mL xylazine (2%; Biowet, Pulawy, Poland) and 2.5 mL ketamine (100 mg mL^−1^; Biowet, Poland). After 10 min, responses to stimuli and corneal reflex were checked. If necessary, another dose of xylazine and ketamine was given. Next, beavers were sacrificed by decapitation under full anesthesia. All efforts were made to minimize animal suffering. During performed vivisections and tissue harvesting, no pathological changes were found in particular/individual organs and tissues.

**Figure 6:**
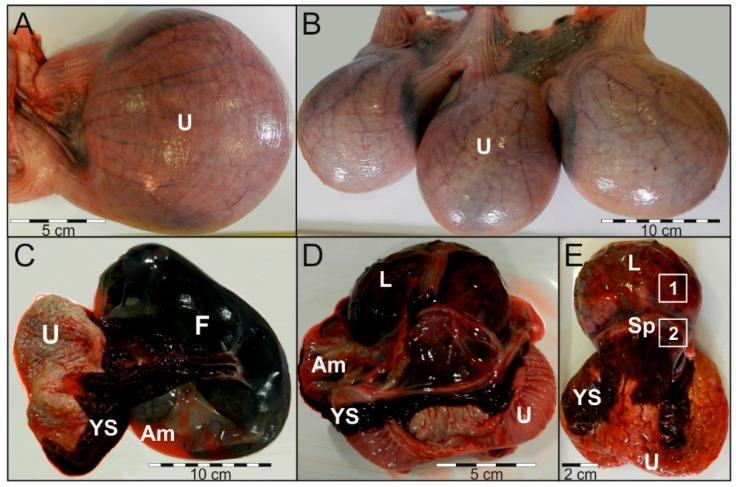
Gross appearance of the beaver gestation sac and placenta. Uterus with single sac (**A**) or triplets (**B**). Gestation sac with: riven yolk sac (**C**) or removed fetus (**D**). External view of the placenta with quads demonstrating regions harvested for: (1) IHC and Western; (2) IHC and RNA isolation (**E**). Abbreviations: U—uterus; F—fetus; YS—yolk sac; Am—amnion; L—labyrinth; Sp—subplacenta.

Placental tissues (*n* = 6) were collected post mortem from adult Cf females (*n* = 3) during single, twin, or triple gestation (Table 2). Harvested placental tissues were separated for discoid (labyrinth zone) and extra-discoid (subplacenta) explants (Figure 6), then immediately preserved in liquid nitrogen or placed in sterile PBS supplemented with penicillin (100 I.U. mL^−1^) and streptomycin (100 μL mL^−1^), transported to the Department of Animal Physiology, stored at −70 °C or subjected to in vitro study.

### 4.3. RNA-seq (cDNA Library Construction, Paired-End Sequencing and Bioinformatics)

Total RNA was isolated from subplacenta explants harvested from single and twin pregnancy (Table 2) using a RNeasy Kit in conjunction with the RNase-Free DNase Set (Qiagen, Hilden, Germany) following the manufacturer’s recommendations. The concentration and purity of total RNA were evaluated via microfluidic electrophoresis (2100 Bioanalyzer, Agilent Technologies, Santa Clara, CA, USA). Total RNA samples with high quality (RIN ≥ 8) were then subjected to complementary DNA (cDNA) library construction.

Each library was constructed according to the protocol of the TruSeq Stranded mRNA LT Sample Prep Kit (Illumina, San Diego, CA, USA). Briefly, the main steps of cDNA library preparation were: RNA purification and fragmentation, synthesis of the first and the second strand of cDNA, 3′ adenylation and adaptor ligation, size-selection, indexing PCR, and finally quantification. The indexed libraries were diluted and pooled in equimolar ratios, then pair-end sequenced and 2 × 100 bp reads were obtained (HiSeq2500, Illumina, San Diego, CA, USA).

To perform quality control of the obtained raw reads, the FASTQC tool was used. To trim out the Illumina adaptors and poly(A) stretches, FLEXBAR software was applied. The data set was filtered to remove reads shorter than 32 bp and PHRED < 10. Trimmed reads were *de novo* assembled with the use of the TRINITY software (Version r20140717, Cambridge, MA, USA). To select potential target sequences, reconstructed contigs were searched for various *AP* members.

### 4.4. Capillary Sequencing

Capillary sequencing was performed to confirm the obtained RNA-Seq coding sequence of the beaver placental *AP*. Briefly, an Enhanced Avian HS RT-PCR Kit (Sigma-Aldrich, St. Louis, MO, USA) was used to transcribe RNA (from the same samples that were used for RNA-Seq) to cDNA in two-step RT-PCR. To synthesize the first strand of cDNA, a mix of dNTPs and random hexamers were used as primers. Furthermore, target cDNA was amplified with specific primers (Table 3) designed by used GENEIOUS R7 and Oligo Calc [50] on *AP* sequence identified in RNA-Seq.

The obtained *AP* amplicons were electrophoresed, gel-out purified and used as cDNA templates for the capillary sequencing (3130 Genetic Analyzer, Applied Biosystems, Foster City, CA, USA) of both strands in sense and anti-sense directions. Labeling was performed with a BigDye Terminator v3.1 Cycle Sequencing Kit (Applied Biosystems, Foster City, CA, USA) according to the manufacturer’s protocol. Labeled amplicons were purified with a BigDye X Terminator Purification Kit (Applied Biosystems, Foster City, CA, USA) and separated in capillaries filled with POP-7™ polymer (Applied Biosystems, Foster City, CA, USA). The identified beaver *AP* cDNA sequence was analyzed using GENEIOUS R7 software. Additionally, in silico analyses of the cDNA were performed with the following online tools [51,52,53].

### 4.5. Cross-Species Specificity of Anti-PAG Polyclonals

The primary rabbit polyvalent anti-porcine PAG polyclonals were raised against native (glycosylated) antigens produced in vitro by porcine chorionic explants (anti-pPAG-P) and recombinant (non-glycosylated) porcine PAG2 antigen (anti-Rec pPAG2) [54,55]. High specificity of the anti-PAG polyclonals was previously defined by homo- and heterologous PAGE/Western immunoblotting of multiple PAG isoforms [11,12]. These polyclonals were previously used for placental aspartic proteinases detection in various species with different placenta type including pig [45], bison [44], alpaca [46], camels [47], and moose [40].

### 4.6. In Vitro Cultures and Western Blotting

Placental explants were long-term cultured (up to 209 h) for the in vitro production of secretory proteins by the method described [11,12]. Briefly, discoid placentas harvested separately for each fetus (*n* = 5) were minced into small pieces (1–3 mm^3^) and washed in PBS that was collected as pre-culture protein fractions (0 h). Explants were cultured in serum-free Dulbecco’s Modified Eagle’s Medium (DMEM) supplemented with antibiotics (ICN, Santa Ana, CA, USA), penicillin (100 I.U./mL), streptomycin (100 μL/mL), and nystatin (240 I.U. mL^−1^), placed in ~200 mL of DMEM in 1.25-liter flasks (37 °C, 5% CO_2_:95% air; IG 150, Jouan, Saint-Herblain, France). During extended periods of the long-term experiments (209 h), collected media were replaced (in 6–12 h intervals) by fresh DMEM.

Secretory placental proteins were isolated from media after long-term culture by ultra-filtration (>10 kDa MWCO; Merc Millipore, Darmstadt, Germany). In addition, cellular proteins were isolated from cultured and frozen placentas that were homogenized and lysed by an alkaline buffer (Total Protein Extraction Kit, Genoplast, Rokocin, Poland). The secretory and cellular protein concentrations were determined using the Bradford procedure.

Beaver proteins (secretory and cellular), porcine placental proteins (positive control), endometrial proteins of cycling pigs (negative control), and a molecular marker (10–250 kDa; Fermentas, Waltham, MA, USA) were parallel separated by SDS-PAGE. After electrophoresis, total protein profiles were visualized by staining polyacrylamide gels with Coomassie Brilliant Blue (CBB). PAGE-separated proteins (duplicates) were transferred onto 0.45 µm nitrocellulose membranes (Optitran BA-S58, Whatman, GE Healthcare Life Science, Pittsburgh, PA, USA) and analyzed by Western blotting. Identification of beaver placental AP fractions was done by heterologous (cross-species) immuno-detection as described previously [54,55]. Briefly, Western blot analysis was performed using primary rabbit anti-pPAG-P (titer 1:200) and secondary mouse anti-rabbit IgG monoclonals–conjugated with alkaline phosphatase in titer 1:100,000 (Sigma-Aldrich, St. Louis, MO, USA). Visualization of the immune complexes was performed with the use of standard substrates (NBT and BCIP) for alkaline phosphatase activity. Photographed gels and blots were archived by GBox (Syngen, Cambridge, UK).

### 4.7. Heterologous Double Fluorescent Immunohistochemistry (htdF-IHC)

Labyrinth and subplacenta explants were cryo-sectioned (–20 °C; 6 μm), fixed, dehydrated, and then subjected to htdF-IHC as previously described [40,44,46,47]. Briefly, the htdF-IHC was performed with primary rabbit anti-pPAG-P (1:300) or anti-Rec pPAG2 (1:50) polyclonals. Parallel negative controls were performed without the primary polyclonals. The PAG immuno-complexes were visualized with secondary goat anti-rabbit polyclonals (1:1000)–conjugated with Alexa 488 fluorophore (green), among all the nuclei of placental cells stained with propidium iodide (red).

## 5. Conclusions

This study provided pioneer data reporting the pep/PAG-L transcript and proteins in the beaver placenta, the largest rodent in Eurasia. Despite the similarity of the pep/PAG-L to previously known APs, this study described novel aspects of this family including placental cDNA as well as production, secretion, and protein profiles. This will have an important influence on the further explanation of the proper and efficient implantation, placenta development, and pregnancy maintenance in this species. These results also extend the present knowledge about the placental transcriptome and proteome that will be helpful to identify mechanisms underlying the course of normal pregnancy across species with discoid placenta.

## Figures and Tables

**Table 1:** Comparison of the aa sequence of N- and C-terminal domains in beaver pep/PAG-L and various AP members.

Protein Name ^a^	N-Domain ^b^	C-Domain ^b^
pep/PAG-L	VLFDTGSSNLWV	GIVDTGTSLLTV
hPepsinogen C	............	A...........
mPepsinogen C	............	..........VM
hPepsinogen A	.V..........	A..........G
mCathepsin D	.V..........	A.........VG
mNapsin A	.V..........	A.L......I.G
mPepsinogen F	.VL.....V...	..M........G
fPAG	.I......D...	A.I.......IG
TrNothepsin	.V......D...	A........IAG
pPAG2, 4, 6, 10	.V......D...	A.......M.HG
oPAG2	.V......D...	AL.......IHG
bPAG2	.V.....A....	ALL.....MIYG
mRenin	.M.....A....	VV....S.FISA
mPepsinogen A	.VL.....V...	..M........G
ePAG	.I.....AD...	A.........LG
zPAG	.I.....AD...	A.........LG
Pf plasmepsin I	FI.....A....	A...S...SI.A
Pr plasmepsin I	FI.....A....	VV..S...SI.A
pPAG1, 3, 5	.I...A..D...	A.L.S.SAF.LG
Pr plasmepsin II	FIL....A....	C...S...AI..
Pf plasmepsin II	FIL....A....	C...S...AI..
Pf histo-AP	FIL....A....	C...S...AI..
Pr histo-AP	F..H.A...V..	V.L.SA..AI..

^a^ b—bovine, e—equine, f—feline, h—human, m—mouse, o—ovine, p—porcine, z—zebra, *Plasmodium falciparum*—Pf, *Plasmodium reichenowi*—Pr, Tr—*Takifubu rubripes*; ^b^ identical aa are dotted.

**Table 2:** Characteristics of the animals and performed analyses.

Animal Code	Age ^a^ [years]	Mass [kg]	Gestation	Fetal Sex	Analyses
#25	1.5–2	14.92	Single	female	RNA-seq; capillary sequencing; IHC; Western
#29	2.5–3	20.22	Twin	29/1 male	IHC
				29/2 female	IHC; Western
#26	>3	22.09	Triple	26/1 female	IHC; Western
				26/2 male	RNA-seq; capillary sequencing; IHC; Western
				26/3 female	IHC; Western

^a^ Age of the Cf females was estimated due to age structure and average body mass of beavers [49]. # number (No.).

**Table 3:** Specific primers applied for the beaver placental AP cDNA amplification.

Ordinal No. of Primer Pairs	Primers Name	Sequence (5′–3′)	Position ^a^	Amplicon Length (bp) ^a^
1	ATG_F	ATGAAGTGGATAGTGGTGGCC	1–21	~1110
	9_R	GAAGACATCWCCMAGGATCCAA	1089–1110	
2	2_F	TTCCTGAAGAMSCACAA	124–140	~436
	5_R	TAGGCCAGSCCCAKGATGCCATC	538–560	
3	3_F	GCTCCTCCAACCTGTGGGT	287–305	~568
	7_R	CAGAGAGGTGCCKGTGTCMACAA	833–855	
4	5_F	GATGGCATCMTGGGSCTGGCCTA	538–560	~572
	9_R	GAAGACATCWCCMAGGATCCAA	1089–1110	
5	7_F	TTGTKGACACMGGCACCTCTCTG	833–855	~424
	3′utr_R	CCAGAGAAGAGGCACAGATAGA	1236–1257	

^a^ Position and amplicon length was estimated due to the beaver placental AP sequence identified using RNA-seq.

## Abbreviations

AP	Aspartic proteinases
cDNA	complementary DNA
Cf	*Castor fiber*
PAG-L	Pregnancy-Associated Glycoprotein-Like family
PAGs	Pregnancy-Associated Glycoproteins
pep/PAG-L	AP identified in the Eurasian beaver placenta
